# How Do Embryos Know Left from Right?

**DOI:** 10.1371/journal.pbio.0030291

**Published:** 2005-07-26

On the outside, humans and other vertebrates seem to be bilaterally symmetrical. Draw a line down your body from the top of your head to your feet, and what is to the left of the line is pretty much the mirror image of what is to the right. But think about your internal organs. Your heart is on the left of your body, while your liver is over to the right. This left-right asymmetry, like the other asymmetries of vertebrate bodies, is established early in embryonic life. The symmetrical ball of cells formed after fertilization of the egg quickly develops a head and tail end (an anteroposterior axis) and a front and back (a dorsoventral axis). Once these identities have been established, the embryo then specifies its left and right sides—an event called left-right symmetry breaking.

Work in early mouse embryos suggests that left-right symmetry breaking arises from the leftward flow of extra-embryonic fluid. This flow is somehow generated by beating cilia, rod-like unicellular organisms through water. During evolution, however, multicellular organisms retained cilia on cells that move extracellular fluid; for example, respiratory tract cells use cilia to flush away bacteria and other debris. And in the embryo, cilia on cells in a region called the node produce the symmetry-breaking leftward flow, or “nodal flow.” As a result, in mouse embryos with mutant cilia that fail to beat, nodal flow is not established, and some mice develop with their internal organs on the wrong side. [Fig pbio-0030291-g001]

**Figure pbio-0030291-g001:**
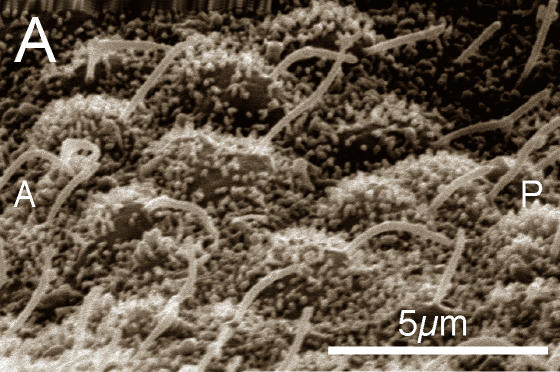
A scanning electron micrograph of mouse embryo node cilia helped researchers determine the cilia's tilt direction, a factor that contributes to embryo asymmetry

But it's not clear how beating nodal cilia actually produce a leftward fluid flow. Unlike most cilia, which beat in a whip-like back-and-forth motion, nodal cilia beat by twirling in a circle; cilia twirling in fluid should act like a propeller and create a vortex (a circular flow of water). But nodal cilia don't produce a vortex; they produce a leftward flow. Models of fluid dynamics suggest that the nodal cilia might be able to do this if they are tilted. And as Hiroshi Hamada and colleagues now report, this is indeed the case; nodal cell cilia are tilted toward the embryo's tail end, rather than sticking straight up out of the plane of the node.

To investigate whether the cilia in mouse embryonic nodes are tilted, the researchers first traced their trajectories using a high-speed camera. Tracing the path of the tip of a straight-up cilium as it beats should yield a circular trace, but the researchers actually recorded elliptical and D-shaped traces. The authors explain that elliptical traces are representative of beating cilia tips viewed at an angle, while the D-shaped traces result when the cilia are tilted so much that they slam into the “floor”—the embryonic cell surface—during their circuit. Scanning electron microscopy then revealed that all the cilia in the node tilted toward the posterior of the embryo and that each cilium was located toward the back of its node cell. Finally, the researchers built a 1,000× scale model of an embryonic node using wires to represent cilia and thick silicone fluid to represent the extra-embryonic fluid. As predicted by theoretical fluid dynamics, the liquid in this model always flowed leftward when the tilted wires were rotated clockwise.

Hamada and colleagues propose that the position of the cilia and their tilt is determined by the pre-existing axis asymmetries (anteroposterior and dorsoventral asymmetries) in the embryo. The tendency of the cilia to rotate clockwise then produces the leftward fluid flow across the node that breaks embryonic left-right symmetry. Whether a similar mechanism occurs in vertebrates other than mice, and how exactly leftward flow contributes to left-right axis establishment remains to be determined, but at least the mystery of how rotating cilia can produce a linear flow has now been solved.

